# Discrepancy in the transmissibility of multidrug-resistant *mycobacterium tuberculosis* in urban and rural areas in China

**DOI:** 10.1080/22221751.2023.2192301

**Published:** 2023-03-29

**Authors:** Meng Li, Liping Lu, Mingcheng Guo, Qi Jiang, Lan Xia, Yuan Jiang, Shu Zhang, Yong Qiu, Chongguang Yang, Yiwang Chen, Jianjun Hong, Xiaoqin Guo, Howard Takiff, Xin Shen, Chuang Chen, Qian Gao

**Affiliations:** a Key Laboratory of Medical Molecular Virology (MOE/NHC/CAMS), School of Basic Medical Science, Shanghai Medical College, Shanghai Institute of Infectious Disease and Biosecurity, Fudan University, Shanghai, People’s Republic of China; b National Clinical Research Center for Infectious Diseases, Shenzhen Third People’s Hospital, Shenzhen, People’s Republic of China; c Department of Tuberculosis Control, Songjiang District Center for Disease Control and Prevention, Shanghai, People’s Republic of China; d Department of Tuberculosis Control, Wusheng County Center for Disease Control and Prevention, Guang’an, People’s Republic of China; e School of Public Health, Renmin Hospital Public Health Research Institute, Wuhan University, Wuhan, People’s Republic of China; f Sichuan Provincial Center for Disease Control and Prevention, Institution for Tuberculosis Prevention and Control, Chengdu, People’s Republic of China; g Tuberculosis Laboratory, Shanghai Municipal Center for Disease Control and Prevention, Shanghai, People’s Republic of China; h School of Public Health (Shenzhen), Shenzhen Campus of Sun Yat-sen University, Shenzhen, People’s Republic of China; i Laboratorio de Genética Molecular, CMBC, IVIC, Caracas, Venezuela

**Keywords:** Multidrug-resistant tuberculosis, transmissibility, whole-genome sequencing, urban and rural China, TB control programmes

## Abstract

The fitness of multidrug-resistant tuberculosis (MDR-TB) is thought to be an important determinant of a strain’s ability to be transmitted. Studies in the laboratory have demonstrated that MDR-TB strains have reduced fitness but the relative transmissibility of MDR-TB versus drug-susceptible (DS) TB strains in human populations remains unresolved. We used data on genomic clustering from our previous molecular epidemiological study in Songjiang (2011-2020) and Wusheng (2009-2020), China, to compare the relative transmissibility of MDR-TB versus DS-TB. Genomic clusters were defined with a threshold distance of 12-single-nucleotide-polymorphisms and the risk for MDR-TB clustering was analyzed by logistic regression. In total, 2212 culture-positive pulmonary TB patients were enrolled in Songjiang and 1289 in Wusheng. The clustering rates of MDR-TB and DS-TB strains were 19.4% (20/103) and 26.3% (509/1936), respectively in Songjiang, and 43.9% (29/66) and 26.0% (293/1128) in Wusheng. The risk of MDR-TB clustering was 2.34 (95% CI 1.38-3.94) times higher than DS-TB clustering in Wusheng and 0.64 (95% CI 0.38-1.06) times lower in Songjiang. Neither lineage 2, compensatory mutations nor rpoB S450L were significantly associated with MDR-TB transmission, and katG S315 T increased MDR-TB transmission only in Wusheng (OR 5.28, 95% CI 1.42-19.21). MDR-TB was not more transmissible than DS-TB in either Songjiang or Wusheng. It appears that the different transmissibility of MDR-TB in Songjiang and Wusheng is likely due to differences in the quality of the local TB control programmes. Suggesting that the most effective way to control MDR-TB is by improving local TB control programmes.

## Introduction

It was originally thought that drug-resistant (DR) mutations in *Mycobacterium tuberculosis* introduce fitness costs [1, 2] that compromise the virulence of the DR strains and hinder their ability to be transmitted and cause secondary cases [3, 4]. Accordingly, mathematical modelling predicted that this reduced fitness would limit the spread of multidrug-resistant tuberculosis (MDR-TB) and minimize its impact on global TB control [5]. These predictions, however, appear to have grossly underestimated the growth and persistence of DR strains, as the MDR-TB epidemic has become a public health issue of global concern [6]. In 2019, there were 464,000 global cases of rifampicin-resistant TB, 78% of which were MDR [7]. Early in the MDR-TB epidemic, most MDR cases likely arose through the secondary acquisition of drug resistance as a result of inadequate therapy of drug-susceptible (DS) TB. Currently, though, the majority of MDR-TB cases are due to the transmission of MDR strains [8–11]. The inconsistency between the theory of lower fitness in DR strains and the reality of the global MDR-TB epidemic has led researchers to explore the proposed fitness effects of DR mutations by comparing the relative transmissibility of MDR-TB versus DS-TB strains.

Although laboratory studies have demonstrated that DR strains have reduced fitness [3, 4], *in vitro* fitness does not provide an accurate representation of bacterial transmissibility. The transmission of *M. tuberculosis* is a complex process influenced by many determinants, including host factors, bacterial factors and socioeconomic status. While it is difficult to control for the non-bacterial variables, population-based epidemiological studies nevertheless provide an *in vivo* measure of the transmissibility of *M. tuberculosis*.

Studies of the transmissibility of MDR-TB in human populations have approached the question in two ways. One approach compares the risk of infection or disease among household contacts of patients with DS-TB versus MDR-TB. The second approach compares the risk of genomic clustering in patients with DS-TB versus those with MDR-TB in the general population [12, 13]. In a small study conducted in Brazil, Teixeira et al. found that the risk of infection and disease among household contacts of patients with DS-TB and MDR-TB was comparable [14]. However, several studies found a greater risk of infection [13, 15] and a lower risk of disease [12] among household contacts of MDR-TB patients. Studies based on molecular epidemiology have also reached contradictory conclusions, with some studies finding that MDR-TB strains were more likely to belong to genomic-clusters than DS strains [16–20], while other studies found less clustering of MDR strains [21–24]. In addition to the discrepancy of the findings, the differences in study designs limit comparisons between these reports, and as a result, the relative transmissibility of MDR-TB versus DS-TB strains remains unresolved.

We previously conducted long-term prospective molecular epidemiological studies of TB in the Songjiang district of Shanghai and the Wusheng County of Sichuan, China [25, 26]. In this report, we analyzed the data on genomic clustering from that study to compare the relative transmissibility of MDR-TB versus DS-TB.

## Methods

### Study design and population

Data for this work was obtained from our previous molecular epidemiological studies conducted in Songjiang and Wusheng. Songjiang is a suburban district of Shanghai, one of the most developed cities in China and a frequent destination for internal migrant workers. As 62% of the total Songjiang population are internal migrants, who contribute approximately 75% of the TB cases in the district, Songjiang is a representative site for epidemiological studies of TB in Chinese cities [25]. Community physicians routinely identify individuals with TB-like symptoms or abnormal chest radiographs and refer them to the TB-designated hospital in Songjiang for diagnosis by sputum smear and culture. The study population was comprised of all culture-positive pulmonary TB patients 15 years or older who were diagnosed between Jan 1, 2011 and December 31, 2020. Whole-genome sequencing (WGS) was performed on all of the cultured isolates. At the time of TB diagnosis we collected demographic, clinical and laboratory information on each patient. The same study design used in Songjiang was also used to investigate TB patients diagnosed in Wusheng, a representative rural county in southwestern China, between July 1, 2009 and December 31, 2020 (Supplementary Figure S1) [26]. The study was approved by the institutional review board of the Institutes of Biomedical Sciences, Fudan University and all enrolled patients provided written informed consent.

### WGS

All clinical strains were re-cultured and sequenced as described [27], and a previously validated pipeline was used to identify single nucleotide polymorphisms (SNPs) [26]. In brief, raw sequence reads were trimmed with Sickle (version 1.33) and aligned to the inferred *M. tuberculosis* complex ancestor sequence [28] using BWA-MEM. SAMtools (version 1.3.1) and Varscan (version 2.3.6) were then used to identify SNPs. Pairwise SNP distances were calculated based on the fixed SNPs with a frequency ≥75%. A genomic cluster was defined as strains differing by ≤12 SNPs, consistent with linkage through recent transmission [11].

Based on the identified SNPs, a phylogeny tree was constructed with RAxML-NG (version 1.0.2) software, using the maximum likelihood method with 100 bootstraps and visualized with Interactive Tree of Life (https://itol.embl.de/). Drug-resistance profiles were predicted for the four first-line drugs based on the mutations reported to be associated with resistance [29]. DS-TB was defined as susceptibility to the four first-line drugs and MDR-TB was defined as resistance to at least isoniazid and rifampicin. Compensatory mutations (CMs) were identified based on the mutations previously reported (Supplementary Table S4) [30, 31].

### Statistical analysis

Non-normal continuous data was expressed as medians and interquartile ranges (IQR), while categorical variables were described using proportions. Differences between groups were tested using the Wilcoxon rank sum test or the chi-square test. Logistic regression was used to calculate the risk of clustering of MDR-TB strains, given as odds ratios (OR) with 95% confidence intervals (CI). Variables with *p*-values less than 0.2 in the univariable analysis were included in the multivariable analysis to calculate the adjusted odd ratios (aOR). OR and 95% CI were also calculated to test the association between bacterial genetic background and increased MDR-TB transmission. Results with a *p*-value less than 0.05 were considered statistically significant. All analyses were performed in Stata version 14.0.

## Results

### General population characteristics

**Figure 1.**
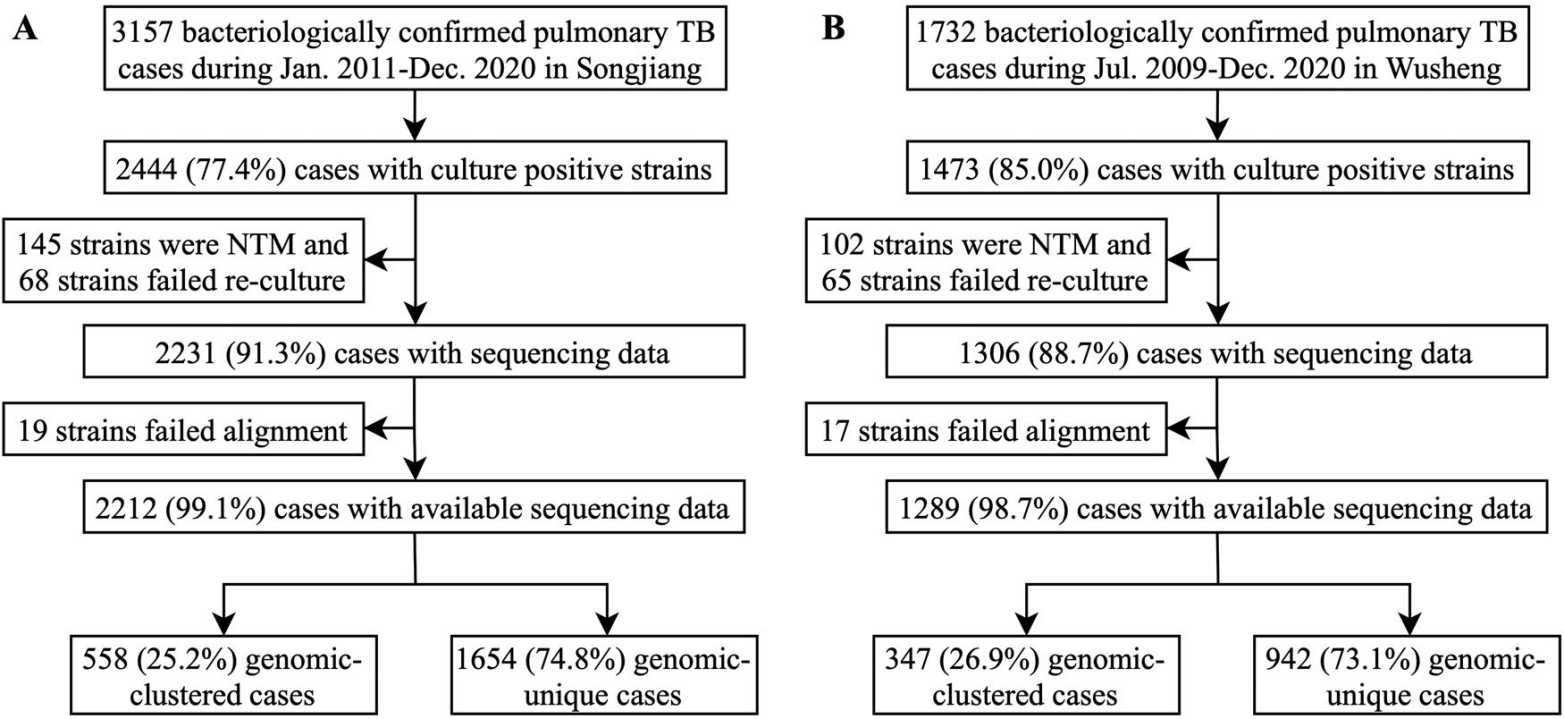
Sample enrolment in Songjiang (A) and Wusheng (B).

Between January 1, 2011 and December 31, 2020, there were 3157 bacteriologically confirmed pulmonary TB patients diagnosed in Songjiang, of which 2444 (77.4%) were culture-positive. After excluding patients whose isolates were non-tuberculous mycobacteria (NTM) and patients whose strains failed re-culture or alignment, a total of 2212 patients were included in the final analysis (Figure 1A). Sample enrolment in Wusheng was described previously [26]. In brief, between July 1, 2009 and December 31, 2020 there were 1473 culture-positive pulmonary TB patients diagnosed in Wusheng, and after excluding patients whose isolates were NTM or whose strains failed re-culture or alignment, a total of 1289 patients were included in the final analysis (Figure 1B). The characteristics of patients are shown in Table 1. Compared with patients in Wusheng, patients in Songjiang were younger (30.0 years vs 50.0 years), had shorter diagnosis delays (3.7 weeks vs 3.9 weeks) and were less likely to be male (69.6% vs 79.0%).

**Table 1.** Characteristics of pulmonary tuberculosis patients in Songjiang and Wusheng.

	Songjiang (%)	Wusheng (%)	*p* value
Total	2212	1289	
Sex			<0.001
Female	673 (30.4)	271 (21.0)	
Male	1539 (69.6)	1018 (79.0)	
Age	30.0 (24.0, 48.0)	50.0 (32.0, 61.0)	<0.001
Occupation			
Farmer	266 (12.0)	1096 (85.0)	<0.001
Others	1946 (88.0)	193 (15.0)	
Internal migrant			–
No	560 (25.3)	–	
Yes	1652 (74.7)	–	
History of tuberculosis			0.069
New cases	2088 (94.4)	1197 (92.9)	
Retreated cases	124 (5.6)	92 (7.1)	
Diagnosis delay/weeks	3.7 (2.1, 6.1)	3.9 (1.3, 5.6)	<0.001
Chest cavitation			0.965
No	1524 (68.9)	889 (69.0)	
Yes	688 (31.1)	400 (31.0)	
Sputum smear status			0.047
Negative	1011 (45.7)	634 (49.2)	
Positive	1201 (54.3)	655 (50.8)	
Drug resistance profile			0.749
Pan-susceptible	1936 (87.5)	1128 (87.5)	
Other DR	173 (7.8)	95 (7.4)	
MDR	103 (4.7)	66 (5.1)	
Beijing strain			<0.001
No	403 (18.2)	523 (40.6)	
Yes	1809 (81.8)	766 (59.4)	

Continuous data were expressed as median (interquartile range) and categorized data as n (%). Comparison between groups were tested by the chi-square test or Wilcoxon rank sum test.

### WGS for genotyping and drug resistance prediction

**Figure 2.**
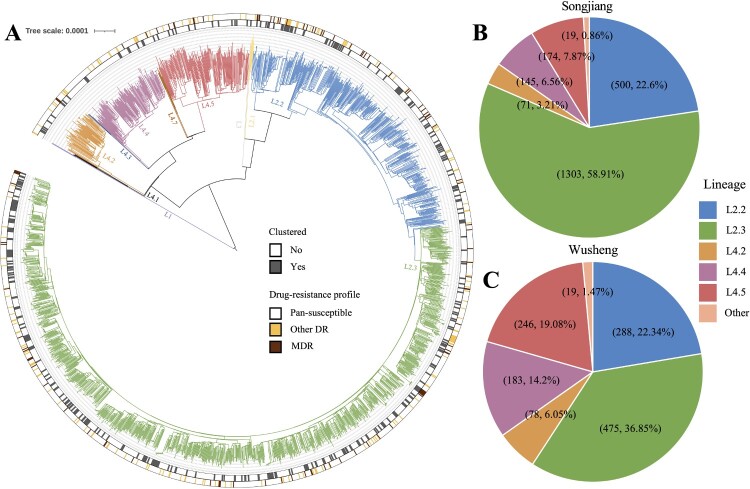
(A) Phylogeny, clustering, and resistance profile of 2212 *Mycobacterium tuberculosis* strains isolated in Songjiang. (B) The sublineage composition of isolates from patients in Songjiang. (C) The sublineage composition of isolates from patients in Wusheng.

The whole genomes of 3501 strains were sequenced with a median depth of 134X (IQR, 101-158) and a median coverage of 99.3% (IQR, 99.2-99.4). Phylogenetic analysis revealed that most strains belonged to Lineage 2 (L2) (Table 1; Figure 2): 81.8% (1809/2212) in Songjiang and 59.4% (766/1289) in Wusheng. Almost all non-L2 strains belonged to L4, predominantly sublineages L4.2, L4.4 and L4.5 (Figure 2). The drug-resistance profiles were similar between the two sites (Table 1). The proportions of DS-TB were 87.5% (1936/2212; 1128/1289) in both Songjiang and Wusheng, and the proportions of MDR-TB were 4.7% (103/2212) and 5.1% (66/1289) respectively.

To identify risk factors associated with the notified MDR-TB patients, we compared the characteristics of MDR and non-MDR patients (Supplementary Table S1). There was a higher proportion of MDR in retreated patients in both sites. In Songjiang, 16.9% (21/124) of the retreated patients and 3.9% (82/2088) of the new patients had MDR-TB, while in Wusheng 16.3% (15/92) and 4.3% (51/1197) of retreated and new patients, respectively, had MDR-TB. In Songjiang there was also a higher proportion of MDR-TB in migrant patients (5.4%, 89/1652) than in patients whose home residence was registered (*hukou*) as Shanghai (2.5%, 14/560).

### Transmission of MDR-TB and its risk factors

To assess the relative transmissibility, we calculated the clustering rates for MDR and DS strains. The clustering rates for MDR and DS strains were 19.4% (20/103) and 26.3% (509/1936), respectively in Songjiang, and 43.9% (29/66) and 26.0% (293/1128) respectively in Wusheng. The median cluster sizes of MDR and DS strains were similar in both Songjiang – 2 (IQR, 2-2) vs 2 (IQR, 2-3), and Wusheng – 2 (IQR, 2-3) vs 2 (IQR, 2-3) (Supplementary Figure S2). We then calculated the aOR for the risk of MDR clustering by adjusting for demographic, clinical and laboratory data (Table 2; Supplementary Table S3). This analysis estimated that in Wusheng the MDR strains were 2.34 (95% CI 1.38-3.94) times more likely to be clustered than DS strains, while in Songjiang the MDR strains were 0.64 (95% CI 0.38-1.06) times less likely to be clustered than DS strains, suggesting that the transmissibility of MDR-TB strains differed in the two sites.

**Table 2.** Multivariable logistic regression of risk factors for clustering in Songjiang and Wusheng.

	Songjiang				Wusheng			
	Non-clustered (%)	Clustered (%)	aOR* (95% CI)	*p* value	Non-clustered (%)	Clustered (%)	aOR^#^ (95% CI)	*p* value
Total	1654	558			942	347		
Sex								
Female	535 (79.5)	138 (20.5)	Ref		196 (72.3)	75 (27.7)	-	
Male	1119 (72.7)	420 (27.3)	1.59 (1.26, 1.99)	<0.001	746 (73.3)	272 (26.7)	-	-
Age								
<25	455 (70.9)	187 (29.1)	4.01 (2.62, 6.15)	<0.001	115 (58.1)	83 (41.9)	2.54 (1.66, 3.88)	<0.001
25–44	681 (73.8)	242 (26.2)	3.38 (2.24, 5.11)	<0.001	248 (75.6)	80 (24.4)	1.16 (0.77, 1.73)	0.472
45–64	298 (78.4)	82 (21.6)	1.92 (1.26, 2.92)	0.002	386 (74.4)	133 (25.6)	1.23 (0.85, 1.78)	0.273
≥65	220 (82.4)	47 (17.6)	Ref		193 (79.1)	51 (20.9)	Ref	
Internal migrant								
No	396 (70.7)	164 (29.3)	Ref		-	-	-	
Yes	1258 (76.2)	394 (23.8)	0.48 (0.37, 0.63)	<0.001	-	-	-	-
Drug resistance profile								
Pan-susceptible	1427 (73.7)	509 (26.3)	Ref		835 (74.0)	293 (26.0)	Ref	
Other DR	144 (83.2)	29 (16.8)	0.57 (0.37, 0.87)	0.008	70 (73.7)	25 (26.3)	1.00 (0.61, 1.62)	0.985
MDR	83 (80.6)	20 (19.4)	0.64 (0.38, 1.06)	0.081	37 (56.1)	29 (43.9)	2.34 (1.38, 3.94)	0.001
Beijing strain								
No	346 (85.9)	57 (14.1)	Ref		397 (75.9)	126 (24.1)	Ref	
Yes	1308 (72.3)	501 (27.7)	2.24 (1.65, 3.03)	<0.001	545 (71.1)	221 (28.9)	1.19 (0.91, 1.54)	0.199

*Adjusted for sex, age, internal migrant, chest cavitation, drug resistance profile and Beijing strain. ^#^Adjusted for age, history of tuberculosis, diagnosis delay, drug resistance profile and Beijing strain.

To identify possible risk factors associated with MDR clustering, we compared the characteristics of clustered and non-clustered MDR patients (Supplementary Table S2). We found that in Wusheng none of the patient characteristics were associated with clustering, while in Songjiang, only gender and diagnostic delay were associated with MDR clustering. In Songjiang, the clustering rate was significantly higher (*p *= 0.019) in male MDR patients (25.7%, 18/70) than in female MDR patients (6.1%, 2/33) and the diagnosis delay was longer in clustered MDR patients (5.0 weeks vs. 2.7 weeks, *p *= 0.0298), but no other patient characteristics were associated with clustering.

**Figure 3.**
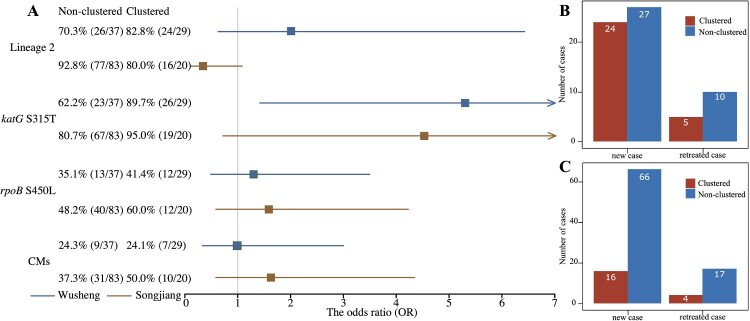
(A) Forest plot of the impact of lineage 2, *katG S315 T*, *rpoB S450L* and compensatory mutations (CMs) on MDR-TB transmission risk. (B) Number of new and retreated MDR-TB cases in Wusheng, stratified by clustered (red) and non-clustered (blue) cases. (C) Number of new and retreated MDR-TB cases in Songjiang, stratified by clustered (red) and non-clustered (blue) cases.

To determine the proportion of MDR-TB cases due to transmission, we separated the new and retreated MDR patients into those with clustered or non-clustered isolates (Figure 3B/C). All clustered MDR patients and non-clustered new MDR patients presumably had primary infections with MDR strains. There were 20 and 29 clustered MDR-TB patients in Songjiang and Wusheng respectively, and 66 and 27, respectively, non-clustered new patients with MDR-TB. Therefore, at least 83.5% (86/103) of MDR patients in Songjiang and 84.8% (56/66) in Wusheng were likely caused by the transmission of MDR strains.

### Impact of bacterial genetic background on MDR-TB transmission

Among the MDR-TB strains in Songjiang and Wusheng, 90.3% (93/103) and 75.8% (50/66), respectively, belonged to L2. Different *M. tuberculosis* lineages are thought to vary in their inherent fitness and it has been suggested that the genetic background of L2 strains can somehow mitigate the fitness costs of rifampicin-resistance mutations [1]. Consistent with this notion, in Wusheng the 48.0% (24/50) clustering rate for L2 strains was higher than the 31.3% (5/16) rate for non-L2 strains. The opposite was found in Songjiang, however, where the 40.0% (4/10) clustering rate for non-L2 strains was more than twice the 17.2% (16/93) clustering rate for L2 strains. When we analyzed the overall association of L2 with MDR clustering, however (Figure 3A), we found no significant correlation of lineage with clustering in either Wusheng (OR 2.03, 95% CI 0.63-6.43) or Songjiang (OR 0.31, 95% CI 0.08-1.15). Previous studies have suggested that L2 strains have an increased capacity for transmission [32], and we also found that patients with an L2 strain had a greater risk of clustering (Table 2). L2 strains did not, however, show an increased rate of MDR clustering.

Compensatory mutations (CMs) are thought to alleviate the fitness costs of some drug-resistant mutations, especially the *rpoB* mutations confering resistance to rifampicin. If CMs restore the fitness MDR strains, they could perhaps augment the transmission success of MDR-TB [33, 34]. We identified a total of 23 different CMs (Supplementary Table S4) that were present in 39.8% (41/103) of the MDR-TB strains in Songjiang and in 24.2% (16/66) of the MDR-TB strains in Wusheng. The clustering rates for MDR-TB strains with and without CMs were 24.4% (10/41) and 16.1% (10/62) in Songjiang, and 43.8% (7/16) and 44.0% (22/50) in Wusheng. Statistical analysis of these proportions (Figure 3A), however, showed that CMs had no significant association with the risk of MDR clustering in either Songjiang (OR 1.68, 95% CI 0.64-4.40) or Wusheng (OR 0.99, 95% CI 0.33-3.00).

In clinical isolates, *rpoB* S450L is the most common mutation conferring rifampicin resistance and *katG* S315 T is the most common mutations conferring isoniazid resistance, and both mutations have been shown to have low *in vitro* fitness costs [2, 35]. The *rpoB* S450L mutation was found in 50.5% (52/103) of MDR-TB strains in Songjiang and 37.9% (25/66) of MDR-TB strains in Wusheng. Although the clustering rates for strains with the *rpoB* S450L mutation were higher than for strains without this mutation in both Songjiang – 23.1% (12/52) vs 15.7% (8/51), and Wusheng – 48.0% (12/25) vs 41.5% (17/41) (Figure 3A), the differences were not significant in either site: Songjiang (OR 1.61, 95% CI 0.61-4.25); Wusheng (OR 1.30, 95% CI 0.49-3.50). This suggests that if the putative low fitness cost of the *rpoB* S450L mutation promotes transmission, the effect is small and would require a larger data set to perhaps reach statistical significance.

A similar analysis was applied to the *katG* S315 T substitution that confers isoniazid resistance. This mutation was found in 83.5% (86/103) and 74.2% (49/66) of MDR-TB strains in Songjiang and Wusheng, respectively, and the clustering rates for strains with and without *katG* S315 T were 53.1% (26/49) and 17.6% (3/17) in Wusheng and 22.1% (19/86) and 5.9% (1/17) in Songjiang. Statistical analysis found that in Wusheng the MDR-TB strains with *katG* S315 T had a significantly increased risk of clustering (Figure 3A) (OR 5.28, 95% CI 1.42-19.21), but there was no association of *katG* S315 T with clustering in Songjiang (OR 4.54, 95% CI 0.71-∞).

## Discussion

In this report we compared the transmissibility of MDR-TB versus DS-TB strains using data from long-term molecular epidemiological studies conducted in Songjiang and Wusheng. We found that the clustering rates for MDR and DS strains were 43.9% and 26.0% in Wusheng and 19.4% and 26.3% in Songjiang, indicating that compared to DS-TB strains, the MDR-TB strains showed greater transmissibility in Wusheng (aOR = 2.34, 95% CI 1.38-3.94) but weaker transmissibility in Songjiang (aOR = 0.64, 95% CI 0.38-1.06). Further analysis showed that neither lineage 2, CMs nor the *rpoB* S450L substitution were significantly associated with MDR-TB transmission, and the *katG* S315 T substitution increased MDR-TB transmission only in Wusheng (OR 5.28, 95% CI 1.42-19.21).

Population-based molecular epidemiological studies have produced conflicting results on the relative transmissibility of MDR versus DS strains. Alland et al. collected 104 strains from patients diagnosed in a New York City hospital during 1989–1992 and found that the clustering risk for MDR-TB strains was 6.5 (95% CI 1.5-33.3) times higher than for DS-TB strains [16]. Similarly, a small study in Archangel Oblast, Russia by Toungoussova et al. collected 119 strains in 1998 and 1999 and found that MDR-TB strains were much more likely to be clustered (OR 9.2, 95% CI 2.4-47.7) [17]. Yang et al. conducted a molecular epidemiological study in five regions in China in 2009–2012 and also found that MDR-TB strains had a greater risk of clustering (OR 1.86, 95% CI 1.25-2.63) than DS-TB strains [18]. In contrast, other studies reported opposite results. Garcia-Garcia et al. collected 326 strains in the Orizaba Health Jurisdiction of Mexico during 1995–1999 and found that MDR-TB strains had a lower risk of clustering (OR 0.31, 95% CI 0.12-0.81) [22]. Godfrey-Faussett et al. conducted a molecular epidemiological study of 371 goldminers with culture-positive TB in South Africa and also found that the MDR-TB patients had a lower risk of clustering (OR 0.27, 95% CI 0.09-0.83) [23]. In a molecular epidemiological study from 2014–2017 in Shenzhen, China, Yang et al. found that MDR-TB and DS-TB strains had similar risks of clustering (OR 0.85, 95% CI 0.46-1.57) [24]. Many of these previous studies were of short duration and had small sample sizes, and the differences in study design and genotyping methods make it difficult to draw comparisons between them. We based our analysis on the results of a 10-year prospective molecular epidemiological study using the same design in both urban Songjiang and rural Wusheng and therefore believe our data faithfully represents the transmissibility of the TB strains and allow meaningful comparisons of the results from the two sites.

At present there is no model that faithfully simulates human TB transmission. This limits our understanding of the relative transmissibility of MDR-TB versus DS-TB and the role of different factors that influence transmission, but epidemiological data can provide useful information. There are many incompletely defined influences affecting MDR-TB transmission, including host factors, bacterial factors, and socioeconomic status. Socioeconomic status is especially complex and difficult to assess quantitatively, but is generally reflected in the quality or effectiveness of the local TB control programmes and its ability to find and cure local TB cases, thereby limiting transmission. We refer to this generically as the quality of the local TB control programmes, which is possible to improve. Because we cannot quantify the efficacy of TB prevention and control programme, we used the exclusion method to analyze the influence of other factors that may affect MDR-TB transmission. Our analysis found that both host (Table 2; Supplementary Table S3) and bacterial factors (Figure 3) had a limited effect on MDR-TB transmission, and therefore believe that the differences in the transmissibility of MDR-TB in Songjiang and Wusheng are mainly due to differences in the quality of the local TB control programmes. Although it is difficult to quantify and compare the effectiveness of local TB prevention and control programmes, our data, derived from two regions with distinct TB prevention and control programmes, provides an opportunity to assess their influence on the transmissibility of MDR-TB strains. Shanghai is one of the most developed cities in China with one of the best TB control programmes and a TB incidence of 26.5 per 100,000 population [36]. Since 2011, Shanghai has implemented an integrated MDR-TB control model that includes MDR-TB patient detection, expert panel consultation on diagnosis, treatment in designated hospitals, case management and medical cost reimbursement [37]. Wusheng is a poor rural area with a TB incidence of 95.8 per 100,000 population [38]. Up until 2021, programmes for the diagnosis, treatment and management of MDR-TB patients were lacking in Wusheng and MDR-TB patients seeking treatment had to travel voluntarily to TB-designated provincial medical institutions far from their homes.

High quality TB prevention and control programmes that diagnose and effectively treat MDR-TB early in the course of the disease can minimize the time that MDR-TB patients are infectious and thereby interrupt MDR-TB transmission. As a result, the clustering rates of MDR and DS strains would be similar, as we found in Shanghai. In contrast, in areas with inadequate TB control programmes, the delay in diagnosing and appropriately treating MDR-TB would leave infectious MDR patients in their community for longer time periods, leading to more transmission. Without the ability to promptly detect MDR-TB, all patients would receive a first-line treatment regimen that would reduce DS-TB transmission but allow continued spread of MDR-TB strains, leading to higher MDR-TB clustering rates.

Although many drug-resistance mutations are thought to engender a vary extent of fitness cost [1–4], other bacterial genetic factors, including belonging to Lineage 2, harbouring the *katG* S315 T and *rpoB* S450L substitutions and having CMs, have been associated with increased or restored bacterial fitness, manifested as an increased risk for genomic clustering [1, 2, 33–35]. Several population-based molecular epidemiological studies have found that the bacterial genetic background was associated with increased MDR-TB transmission. Gygli et al. analyzed MDR-TB strains isolated in Georgia from 2011–2016 and found that strains carrying CMs had greater transmissibility (OR 1.34, 95% CI 1.05-1.71) [31] than those without CMs. Nonghanphithak et al. analyzed DR strains isolated in Thailand from 2014–2017 and found that strains with the *katG* S315 T (OR 3.90, 95% CI 2.46-6.18) or *rpoB* S450L (OR 3.29, 95% CI 2.32-4.66) substitutions had greater transmissibility [39]. These results, however, could reflect specific local circumstances and may not apply to all situations. We recently analyzed global data sets to assess the influence of these bacterial factors on MDR-TB transmission and found that only the *katG* S315 T substitution facilitates MDR-TB transmission, and this association was significant solely in developing countries and regions with high TB burdens [40]. The current report again shows that the bacterial genetic background appears to have a limited effect on MDR-TB transmission, but also confirmed that *katG* S315 T can facilitate MDR-TB transmission in a high TB-burden rural setting with an inadequate control programme.

It is undeniable that the bacterial genetic background has a significant impact on TB transmission in regions where the specific environments and conditions lead large clusters of single clones [31, 39]. For reasons that are undefined, however, no large single-clone clusters have been described in China [41], suggesting that in China the bacterial genetic background is perhaps less important than in other settings.

While a favourable genomic background may increase the transmissibility of some MDR-TB strains, this advantage may not have the opportunity to be manifested if MDR-TB is promptly diagnosed and appropriately treated by an effective TB control programme. An inadequate TB control programme, however, may provide conditions in which the advantageous bacterial genetic factors can promote transmission, which would be lead to increased clustering rates. In our study this was seen with *katG* S315 T isoniazid resistance substitution, which increased MDR-TB transmission in the setting of inadequate TB control in Wusheng but not with the more effective TB control in Songjiang. Transmission is a complex process but the bacterial genetic background does not appear to be the main determinant of MDR-TB transmissibility and clustering, at least in China.

The major limitation of our study is that, despite its 10-year duration, the populations covered and the total number of MDR-TB patients are relatively small. In addition we did not comprehensively analyze and could not quantify the efficacies of the TB prevention and control programmes and their effects on MDR-TB transmission. Our characterization of TB control is based on the TB incidence rates at the two sites and the control measures that have been implemented. Because our study included both urban and rural settings, we believe our results are likely applicable to all of China, but may not be applicable to other settings, especially areas with a high TB incidence, large MDR outbreaks and poor TB control.

In conclusion, MDR-TB strains do not appear to be more transmissible than DS-TB strains in Songjiang and Wusheng. The effect of the host and bacterial factors on MDR-TB transmission appears to be limited and less important than the quality of the local TB control programmes. Our results imply that the most effective way to interrupt MDR-TB transmission and reduce the global incidence of MDR-TB is by improving the diagnosis, treatment and management of MDR-TB patients.

## Supplementary Material

Supplemental Material

## Data Availability

The datasets used and analyzed during the current study are not accessible online, but may be made available upon written request to the corresponding author. Sequencing data were deposited in the Genome Sequence Archive (https://bigd.big.ac.cn/gsa) under BioProject PRJCA008815 and PRJCA010372.

## References

[1] Gagneux S, Long CD, Small PM, et al. The competitive cost of antibiotic resistance in mycobacterium tuberculosis. Science. 2006 Jun 30;312(5782):1944–1946. 16809538 10.1126/science.1124410

[2] Gygli SM, Borrell S, Trauner A, et al. Antimicrobial resistance in mycobacterium tuberculosis: mechanistic and evolutionary perspectives. FEMS Microbiol Rev. 2017 May 1;41(3):354–373. 28369307 10.1093/femsre/fux011

[3] Cohen T, Sommers B, Murray M. The effect of drug resistance on the fitness of mycobacterium tuberculosis. Lancet Infect Dis. 2003 Jan;3(1):13–21. 12505028 10.1016/s1473-3099(03)00483-3

[4] Luciani F, Sisson SA, Jiang H, et al. The epidemiological fitness cost of drug resistance in mycobacterium tuberculosis. Proc Natl Acad Sci USA. 2009 Aug 25;106(34):14711–5. 19706556 10.1073/pnas.0902437106 PMC2732896

[5] Dye C, Williams BG, Espinal MA, et al. Erasing the world’s slow stain: strategies to beat multidrug-resistant tuberculosis. Science. 2002 Mar 15;295(5562):2042–2046. 11896268 10.1126/science.1063814

[6] Lange C, Dheda K, Chesov D, et al. Management of drug-resistant tuberculosis. Lancet. 2019 Sep 14;394(10202):953–966. 31526739 10.1016/S0140-6736(19)31882-3 PMC11524526

[7] WHO. Global tuberculosis report 2020. Geneva: World Health Organization; 2020.

[8] Casali N, Nikolayevskyy V, Balabanova Y, et al. Evolution and transmission of drug-resistant tuberculosis in a Russian population. Nat Genet. 2014 Mar;46(3):279–286. 24464101 10.1038/ng.2878 PMC3939361

[9] Eldholm V, Monteserin J, Rieux A, et al. Four decades of transmission of a multidrug-resistant mycobacterium tuberculosis outbreak strain. Nat Commun. 2015 May 11;6:7119. 25960343 10.1038/ncomms8119 PMC4432642

[10] McBryde ES, Meehan MT, Doan TN, et al. The risk of global epidemic replacement with drug-resistant mycobacterium tuberculosis strains. Int J Infect Dis. 2017 Mar;56:14–20. 28163165 10.1016/j.ijid.2017.01.031

[11] Yang C, Luo T, Shen X, et al. Transmission of multidrug-resistant mycobacterium tuberculosis in Shanghai, China: a retrospective observational study using whole-genome sequencing and epidemiological investigation. Lancet Infect Dis. 2017 Mar;17(3):275–284. 27919643 10.1016/S1473-3099(16)30418-2 PMC5330813

[12] Grandjean L, Gilman RH, Martin L, et al. Transmission of multidrug-resistant and drug-susceptible tuberculosis within households: a prospective cohort study. PLoS Med. 2015 Jun;12(6):e1001843, discussion e1001843. 26103620 10.1371/journal.pmed.1001843 PMC4477882

[13] Becerra MC, Huang CC, Lecca L, et al. Transmissibility and potential for disease progression of drug resistant mycobacterium tuberculosis: prospective cohort study. Br Med J. 2019 Oct 24;367:l5894. 31649017 10.1136/bmj.l5894 PMC6812583

[14] Teixeira L, Perkins MD, Johnson JL, et al. Infection and disease among household contacts of patients with multidrug-resistant tuberculosis. Int J Tuberc Lung Dis. 2001 Apr;5(4):321–328. 11334250

[15] Fox GJ, Anh NT, Nhung NV, et al. Latent tuberculous infection in household contacts of multidrug-resistant and newly diagnosed tuberculosis. Int J Tuberc Lung Dis. 2017 Mar 1;21(3):297–302. 28225339 10.5588/ijtld.16.0576

[16] Alland D, Kalkut GE, Moss AR, et al. Transmission of tuberculosis in New York city. An analysis by DNA fingerprinting and conventional epidemiologic methods. N Engl J Med. 1994 Jun 16;330(24):1710–1716. 7993412 10.1056/NEJM199406163302403

[17] Toungoussova OS, Sandven P, Mariandyshev AO, et al. Spread of drug-resistant mycobacterium tuberculosis strains of the Beijing genotype in the archangel oblast, Russia. J Clin Microbiol. 2002 Jun;40(6):1930–1937. 12037045 10.1128/JCM.40.6.1930-1937.2002 PMC130821

[18] Yang C, Shen X, Peng Y, et al. Transmission of mycobacterium tuberculosis in China: a population-based molecular epidemiologic study. Clin Infect Dis. 2015 Jul 15;61(2):219–227. 25829000 10.1093/cid/civ255 PMC4490233

[19] Li M, Guo MC, Qiu Y, et al. Transmission characteristics of student tuberculosis: a 12-year prospective population-based genomic epidemiological study. Chin J Tuberc Respir Dis. 2023 Jan 12;46(1):19–26. 10.3760/cma.j.cn112147-20220725-00624 36617924

[20] Yang C, Sobkowiak B, Naidu V, et al. Phylogeography and transmission of M. tuberculosis in Moldova: a prospective genomic analysis. PLoS Med. 2022 Feb;19(2):e1003933. 35192619 10.1371/journal.pmed.1003933 PMC8903246

[21] Saavedra Cervera B, Lopez MG, Chiner-Oms A, et al. Fine-grain population structure and transmission patterns of mycobacterium tuberculosis in southern Mozambique, a high TB/HIV burden area. Microb Genom. 2022 Jul;8(7):mgen000844. 35787782 10.1099/mgen.0.000844 PMC9455694

[22] Garcia-Garcia ML, Jimenez-Corona ME, Ponce-de-Leon A, et al. Mycobacterium tuberculosis drug resistance in a suburban community in southern Mexico. Int J Tuberc Lung Dis. 2000 Dec;4(12 Suppl 2):S168–S170. 11144548

[23] Godfrey-Faussett P, Sonnenberg P, Shearer SC, et al. Tuberculosis control and molecular epidemiology in a South African gold-mining community. Lancet. 2000 Sep 23;356(9235):1066–1071. 11009142 10.1016/s0140-6736(00)02730-6

[24] Yang T, Wang Y, Liu Q, et al. A population-based genomic epidemiological study of the source of tuberculosis infections in an emerging city: Shenzhen, China. Lancet Reg Health-W. 2021 Mar;8:100106. 10.1016/j.lanwpc.2021.100106 PMC8315418 34327429

[25] Yang C, Lu L, Warren JL, et al. Internal migration and transmission dynamics of tuberculosis in Shanghai, China: an epidemiological, spatial, genomic analysis. Lancet Infect Dis. 2018 Jul;18(7):788–795. 29681517 10.1016/S1473-3099(18)30218-4 PMC6035060

[26] Li M, Guo M, Peng Y, et al. High proportion of tuberculosis transmission among social contacts in rural China: a 12-year prospective population-based genomic epidemiological study. Emerg Microbes Infect. 2022 Dec;11(1):2102–2111. 35950916 10.1080/22221751.2022.2112912 PMC9448380

[27] Jiang Q, Liu Q, Ji L, et al. Citywide transmission of multidrug-resistant tuberculosis under China’s rapid urbanization: a retrospective population-based genomic spatial epidemiological study. Clin Infect Dis. 2020 Jun 24;71(1):142–151. 31504306 10.1093/cid/ciz790 PMC8127054

[28] Comas I, Coscolla M, Luo T, et al. Out-of-Africa migration and neolithic coexpansion of mycobacterium tuberculosis with modern humans. Nat Genet. 2013 Oct;45(10):1176–1182. 23995134 10.1038/ng.2744 PMC3800747

[29] Papaventsis D, Casali N, Kontsevaya I, et al. Whole genome sequencing of mycobacterium tuberculosis for detection of drug resistance: a systematic review. Clin Microbiol Infect. 2017 Feb;23(2):61–68. 27665704 10.1016/j.cmi.2016.09.008

[30] Liu Q, Zuo T, Xu P, et al. Have compensatory mutations facilitated the current epidemic of multidrug-resistant tuberculosis? Emerg Microbes Infect. 2018 Jun 6;7(1):98. 29872078 10.1038/s41426-018-0101-6 PMC5988693

[31] Gygli SM, Loiseau C, Jugheli L, et al. Prisons as ecological drivers of fitness-compensated multidrug-resistant mycobacterium tuberculosis. Nat Med. 2021 Jul;27(7):1171–1177. 34031604 10.1038/s41591-021-01358-x PMC9400913

[32] Yang C, Luo T, Sun G, et al. Mycobacterium tuberculosis Beijing strains favor transmission but not drug resistance in China. Clin Infect Dis. 2012 Nov;55(9):1179–1187. 22865872 10.1093/cid/cis670 PMC3466095

[33] Handel A, Regoes RR, Antia R. The role of compensatory mutations in the emergence of drug resistance. PLoS Comput Biol. 2006 Oct;2(10):e137. 17040124 10.1371/journal.pcbi.0020137 PMC1599768

[34] Comas I, Borrell S, Roetzer A, et al. Whole-genome sequencing of rifampicin-resistant mycobacterium tuberculosis strains identifies compensatory mutations in RNA polymerase genes. Nat Genet. 2011 Dec 18;44(1):106–110. 22179134 10.1038/ng.1038 PMC3246538

[35] Alame Emane AK, Guo X, Takiff HE, et al. Highly transmitted M. tuberculosis strains are more likely to evolve MDR/XDR and cause outbreaks, but what makes them highly transmitted? Tuberculosis (Edinb). 2021 Jul;129:102092. 34102584 10.1016/j.tube.2021.102092

[36] Shen X, Yang C, Wu J, et al. Recurrent tuberculosis in an urban area in China: relapse or exogenous reinfection? Tuberculosis (Edinb). 2017 Mar;103:97–104. 28237039 10.1016/j.tube.2017.01.007 PMC5638046

[37] Wu Z, Zhang Q, Zhang Z, et al. Effectiveness of an integrated multi-drug resistant pulmonary tuberculosis control model in Shanghai, China. Chin J Antituberc. 2015;37(11):1118–1125.

[38] Li M, Qiu Y, Guo M, et al. Investigation on the cause of recurrent tuberculosis in a rural area in China using whole-genome sequencing: a retrospective cohort study. Tuberculosis (Edinb). 2022 Mar;133:102174. 35124543 10.1016/j.tube.2022.102174

[39] Nonghanphithak D, Chaiprasert A, Smithtikarn S, et al. Clusters of drug-resistant mycobacterium tuberculosis detected by whole-genome sequence analysis of nationwide sample, Thailand, 2014-2017. Emerg Infect Dis. 2021 Mar;27(3):813–822. 33622486 10.3201/eid2703.204364 PMC7920678

[40] Chen Y, Liu Q, Takiff HE, et al. Comprehensive genomic analysis of mycobacterium tuberculosis reveals limited impact of high-fitness genotypes on MDR-TB transmission. J Infect. 2022 Jul;85(1):49–56. 35588941 10.1016/j.jinf.2022.05.012

[41] Zhou Y, Anthony R, Wang S, et al. The epidemic of multidrug resistant tuberculosis in China in historical and phylogenetic perspectives. J Infect. 2020 Apr;80(4):444–453. 31972213 10.1016/j.jinf.2019.11.022

