# Supplementary material for: Discrepancy in the transmissibility of multidrug-resistant mycobacterium tuberculosis in urban and rural areas in China
Source: Emerg Microbes Infect. 2023 Mar 29;12(1):2192301. doi: 10.1080/22221751.2023.2192301 (PMC10062220; doi:10.1080/22221751.2023.2192301)
Supplement: Supplemental Material [file TEMI_A_2192301_SM3865.docx]

**Table S1**. Characteristics of MDR-TB and non-MDR-TB patients in Songjiang and Wusheng.

|  | **Songjiang** | | | **Wusheng** | | |
| --- | --- | --- | --- | --- | --- | --- |
|  | **Non-MDR (N=2109)** | **MDR (N=103)** | **p value** | **Non-MDR (N=1223)** | **MDR (N=66)** | **p value** |
| Sex |  |  | 0.715 |  |  | 0.969 |
| Female | 640 (30.3) | 33 (32.0) |  | 257 (21.0) | 14 (21.2) |  |
| Male | 1469 (69.7) | 70 (68.0) |  | 966 (79.0) | 52 (78.8) |  |
| Age | 30 (24, 49) | 28 (22, 42) | 0.0229^*^ | 50 (32, 62) | 47 (36, 57) | 0.2919^*^ |
| Internal migrants |  |  | 0.005 |  |  | - |
| No | 546 (25.9) | 14 (13.6) |  | - | - |  |
| Yes | 1563 (74.1) | 89 (86.4) |  | - | - |  |
| History of tuberculosis |  |  | <0.001 |  |  | <0.001 |
| New cases | 2006 (95.1) | 82 (79.6) |  | 1146 (93.7) | 51 (77.3) |  |
| Retreated cases | 103 (4.9) | 21 (20.4) |  | 77 (6.3) | 15 (22.7) |  |
| Diagnosis delay |  |  | 0.046 |  |  | 0.487 |
| <2 weeks | 449 (21.3) | 31 (30.1) |  | 411 (33.6) | 17 (25.8) |  |
| 2-4 weeks | 665 (31.5) | 32 (31.1) |  | 212 (17.3) | 15 (22.7) |  |
| 4-8 weeks | 597 (28.3) | 30 (29.1) |  | 350 (28.6) | 21 (31.8) |  |
| ≥8 weeks | 398 (18.9) | 10 (9.7) |  | 250 (20.4) | 13 (19.7) |  |
| Cavitation |  |  | 0.051 |  |  | 0.217 |
| No | 1462 (69.3) | 62 (60.2) |  | 848 (69.3) | 41 (62.1) |  |
| Yes | 647 (30.7) | 41 (39.8) |  | 375 (30.7) | 25 (37.9) |  |
| Sputum smear status |  |  | 0.152 |  |  | 0.698 |
| Negative | 971 (46.0) | 40 (38.8) |  | 600 (49.1) | 34 (51.5) |  |
| Positive | 1138 (54.0) | 63 (61.2) |  | 623 (50.9) | 32 (48.5) |  |

^*^ Wilcoxon non-parametric rank sum test.

**Table S2**. Characteristics of clustered and non-clustered MDR-TB patients in Songjiang and Wusheng.

|  | **Songjiang** | | | **Wusheng** | | |
| --- | --- | --- | --- | --- | --- | --- |
|  | **Non-clustered (N=83)** | **Clustered (N=20)** | **p value** | **Non-clustered (N=37)** | **Clustered (N=29)** | **p value** |
| Sex |  |  | 0.019 |  |  | 0.485 |
| Female | 31 (37.3) | 2 (10.0) |  | 9 (24.3) | 5 (17.2) |  |
| Male | 52 (62.7) | 18 (90.0) |  | 28 (75.7) | 24 (82.8) |  |
| Age | 28 (22, 41) | 28 (24, 54) | 0.4602^*^ | 45 (31, 56) | 48 (41, 57) | 0.2776^*^ |
| Internal migrants |  |  | 0.465 |  |  | - |
| No | 10 (12.0) | 4 (20.0) |  | - | - |  |
| Yes | 73 (88.0) | 16 (80.0) |  | - | - |  |
| History of tuberculosis |  |  | 1.00 |  |  | 0.346 |
| New cases | 66 (79.5) | 16 (80.0) |  | 27 (73.0) | 24 (82.8) |  |
| Retreated cases | 17 (20.5) | 4 (20.0) |  | 10 (27.0) | 5 (17.2) |  |
| Diagnosis delay/weeks | 2.7 (1.6, 4.9) | 5.0 (2.7, 7.4) | 0.0298 | 4.3 (1.9, 5.4) | 3.9 (2.0, 4.9) | 0.933 |
| Chest cavitation |  |  | 0.597 |  |  | 0.123 |
| No | 51 (61.4) | 11 (55.0) |  | 26 (70.3) | 15 (51.7) |  |
| Yes | 32 (38.6) | 9 (45.0) |  | 11 (29.7) | 14 (48.3) |  |
| Sputum smear status |  |  | 0.695 |  |  | 0.641 |
| Negative | 33 (39.8) | 7 (35.0) |  | 20 (54.1) | 14 (48.3) |  |
| Positive | 50 (60.2) | 13 (65.0) |  | 17 (45.9) | 15 (51.7) |  |
| Beijing strain |  |  | 0.1 |  |  | 0.24 |
| No | 6 (7.2) | 4 (20.0) |  | 11 (29.7) | 5 (17.2) |  |
| Yes | 77 (92.8) | 16 (80.0) |  | 26 (70.3) | 24 (82.8) |  |

^*^ Wilcoxon non-parametric rank sum test.

**Table S3**. Univariate logistic regression of risk factors for clustering in Songjiang and Wusheng.

|  | **Songjiang** | | | | **Wusheng** | | | |
| --- | --- | --- | --- | --- | --- | --- | --- | --- |
|  | **Non-clustered (N=1654)** | **Clustered (N=558)** | **OR (95% CI)** | **p value** | **Non-clustered (N=942)** | **Clustered (N=347)** | **OR (95% CI)** | **p value** |
| Sex |  |  |  |  |  |  |  |  |
| Female | 535 (79.5) | 138 (20.5) | Ref |  | 196 (72.3) | 75 (27.7) | Ref |  |
| Male | 1119 (72.7) | 420 (27.3) | 1.46 (1.17, 1.81) | 0.001 | 746 (73.3) | 272 (26.7) | 0.95 (0.71, 1.29) | 0.752 |
| Age |  |  |  |  |  |  |  |  |
| <25 | 455 (70.9) | 187 (29.1) | 1.92 (1.34, 2.75) | <0.001 | 115 (58.1) | 83 (41.9) | 2.73 (1.80, 4.15) | <0.001 |
| 25–44 | 681 (73.8) | 242 (26.2) | 1.66 (1.18, 2.35) | 0.004 | 248 (75.6) | 80 (24.4) | 1.22 (0.82, 1.82) | 0.326 |
| 45–64 | 298 (78.4) | 82 (21.6) | 1.29 (0.86, 1.92) | 0.213 | 386 (74.4) | 133 (25.6) | 1.30 (0.90, 1.88) | 0.155 |
| ≥65 | 220 (82.4) | 47 (17.6) | Ref |  | 193 (79.1) | 51 (20.9) | Ref |  |
| Internal migrant |  |  |  |  |  |  |  |  |
| No | 396 (70.7) | 164 (29.3) | Ref |  | - | - | - | - |
| Yes | 1258 (76.2) | 394 (23.8) | 0.76 (0.61, 0.94) | 0.011 | - | - | - |  |
| History of tuberculosis | |  |  |  |  |  |  |  |
| New cases | 1558 (74.6) | 530 (25.4) | Ref |  | 869 (72.6) | 328 (27.4) | Ref |  |
| Retreated cases | 96 (77.4) | 28 (22.6) | 0.86 (0.56, 1.32) | 0.485 | 73 (79.3) | 19 (20.7) | 0.69 (0.41, 1.16) | 0.162 |
| Diagnosis delay |  |  |  |  |  |  |  |  |
| <2 weeks | 363 (75.6) | 117 (24.4) | Ref |  | 309 (72.2) | 119 (27.8) | Ref |  |
| 2-4 weeks | 524 (75.2) | 173 (24.8) | 1.02 (0.78, 1.34) | 0.862 | 163 (71.8) | 64 (28.2) | 1.02 (0.71, 1.46) | 0.916 |
| 4-8 weeks | 456 (72.7) | 171 (27.3) | 1.16 (0.89, 1.53) | 0.276 | 267 (72.0) | 104 (28.0) | 1.01 (0.74, 1.38) | 0.943 |
| ≥8 weeks | 311 (76.2) | 97 (23.8) | 0.97 (0.71, 1.32) | 0.835 | 203 (77.2) | 60 (22.8) | 0.77 (0.54, 1.10) | 0.147 |
| Chest cavitation |  |  |  |  |  |  |  |  |
| No | 1124 (73.8) | 400 (26.2) | Ref |  | 655 (73.7) | 234 (26.3) | Ref |  |
| Yes | 530 (77.0) | 158 (23.0) | 0.84 (0.68, 1.03) | 0.1 | 287 (71.8) | 113 (28.3) | 1.10 (0.85, 1.43) | 0.47 |
| Sputum smear status | |  |  |  |  |  |  |  |
| Negative | 750 (74.2) | 261 (25.8) | Ref |  | 470 (74.1) | 164 (25.9) | Ref |  |
| Positive | 904 (75.3) | 297 (24.7) | 0.94 (0.78, 1.14) | 0.558 | 472 (72.1) | 183 (27.9) | 1.11 (0.87, 1.42) | 0.402 |
| Drug resistance profile | |  |  |  |  |  |  |  |
| Pan-susceptible | 1427 (73.7) | 509 (26.3) | Ref |  | 835 (74.0) | 293 (26.0) | Ref |  |
| Other DR | 144 (83.2) | 29 (16.8) | 0.56 (0.37, 0.85) | 0.006 | 70 (73.7) | 25 (26.3) | 1.02 (0.63, 1.64) | 0.942 |
| MDR | 83 (80.6) | 20 (19.4) | 0.68 (0.41, 1.11) | 0.123 | 37 (56.1) | 29 (43.9) | 2.23 (1.35, 3.70) | 0.002 |
| Beijing strain |  |  |  |  |  |  |  |  |
| No | 346 (85.9) | 57 (14.1) | Ref |  | 397 (75.9) | 126 (24.1) | Ref |  |
| Yes | 1308 (72.3) | 501 (27.7) | 2.33 (1.73, 3.13) | <0.001 | 545 (71.1) | 221 (28.9) | 1.28 (0.99, 1.65) | 0.059 |

DR, drug resistance; MDR, multidrug resistance.

**Table S4**. List of putative compensatory mutations in the *rpoA*, *rpoB*, and *rpoC* genes identified in this study.

| **Gene** | **AA Substitution** | **Genomic Position** | **Ref. Allele** | **Alt. Allele** |
| --- | --- | --- | --- | --- |
| rpoA_Rv3457c | T181A | 3877967 | T | C |
| rpoA_Rv3457c | V183G | 3877960 | A | C |
| rpoA_Rv3457c | T187A | 3877949 | T | C |
| rpoB_Rv0667 | M434V | 761106 | A | G |
| rpoB_Rv0667 | N437D | 761115 | A | G |
| rpoB_Rv0667 | P45R | 759940 | C | G |
| rpoB_Rv0667 | R827C | 762285 | C | T |
| rpoC_Rv0668 | G332S | 764363 | G | A |
| rpoC_Rv0668 | G433S | 764666 | G | A |
| rpoC_Rv0668 | V483G | 764817 | T | G |
| rpoC_Rv0668 | V483A | 764817 | T | C |
| rpoC_Rv0668 | L516P | 764916 | T | C |
| rpoC_Rv0668 | I885V | 766022 | A | G |
| rpoC_Rv0668 | P1040S | 766487 | C | T |
| rpoC_Rv0668 | P1040R | 766488 | C | G |
| rpoC_Rv0668 | V1252M | 767123 | G | A |
| rpoC_Rv0668 | G332R | 764363 | G | C |
| rpoC_Rv0668 | V431M | 764660 | G | A |
| rpoC_Rv0668 | K445R | 764703 | A | G |
| rpoC_Rv0668 | F452C | 764724 | T | G |
| rpoC_Rv0668 | I491V | 764840 | A | G |
| rpoC_Rv0668 | G519D | 764925 | G | A |
| rpoC_Rv0668 | V1252L | 767123 | G | T |

**Figure S1**. Map of China showing the distribution of the tuberculosis patients whose isolates of *Mycobacterium tuberculosis* were included in the study. The selected study field sites were Wusheng, Sichuan and Songjiang, Shanghai. The markers on the map indicate the relative location of the two study sites.

**Figure S2**. Cluster sizes of MDR and drug-susceptible TB strains.
